# Exploring the fatty acid amide hydrolase and cyclooxygenase inhibitory properties of novel amide derivatives of ibuprofen

**DOI:** 10.1080/14756366.2020.1743283

**Published:** 2020-03-23

**Authors:** Alessandro Deplano, Jessica Karlsson, Mona Svensson, Federica Moraca, Bruno Catalanotti, Christopher J. Fowler, Valentina Onnis

**Affiliations:** a Unit of Pharmaceutical, Pharmacological and Nutraceutical Sciences, Department of Life and Environmental Sciences, University of Cagliari, Cagliari, Italy; b Department of Integrative Medical Biology, Umeå University, Umeå, Sweden; c Department of Pharmacy, University of Napoli Federico II, Napoli, Italy

**Keywords:** Ibuprofen amides, FAAH inhibition, fatty acid amide hydrolase, endocannabinoid, cyclooxygenase

## Abstract

Inhibition of fatty acid amide hydrolase (FAAH) reduces the gastrointestinal damage produced by non-steroidal anti-inflammatory agents such as sulindac and indomethacin in experimental animals, suggesting that a dual-action FAAH-cyclooxygenase (COX) inhibitor could have useful therapeutic properties. Here, we have investigated 12 novel amide analogues of ibuprofen as potential dual-action FAAH/COX inhibitors. *N*-(3-Bromopyridin-2-yl)−2-(4-isobutylphenyl)propanamide (Ibu-AM68) was found to inhibit the hydrolysis of [^3^H]anandamide by rat brain homogenates by a reversible, mixed-type mechanism of inhibition with a K_i_ value of 0.26 µM and an α value of 4.9. At a concentration of 10 µM, the compound did not inhibit the cyclooxygenation of arachidonic acid by either ovine COX-1 or human recombinant COX-2. However, this concentration of Ibu-AM68 greatly reduced the ability of the COX-2 to catalyse the cyclooxygenation of the endocannabinoid 2-arachidonoylglycerol. It is concluded that Ibu-AM68 is a dual-acting FAAH/substrate-selective COX inhibitor.

## Introduction

The non-steroidal anti-inflammatory agents (NSAIDs) such as ibuprofen, naproxen and diclofenac have widespread usage around the world, but their use is hampered by the incidence of severe gastrointestinal side effects. The elderly have a high consumption of NSAIDs, and this has resulted in a high incidence of NSAID-related hospitalisations and deaths^1^. There is thus much to be gained by the discovery and development of compounds that are as efficacious as the NSAIDs, but which lack these deleterious gastrointestinal effects.

In a key study from 2009, Naidu, Lichtman and colleagues^2^ reported that the ulcerogenic potency of the NSAID diclofenac was lower in mice lacking the enzyme fatty acid amide hydrolase (FAAH) than in the corresponding wild-type mice. A similar result was found in wild-type mice pre-treated with the irreversible FAAH inhibitor URB597 ((3′-(aminocarbonyl)[1,1′-biphenyl] − 3-yl)-cyclohexylcarbamate). Further, URB597 and diclofenac acted synergistically in reducing acetic acid-induced visceral nociception^2^. FAAH catalyses the hydrolysis of the endogenous cannabinoid (endocannabinoid) ligand anandamide (AEA, arachidonoylethanolamide)^3^ and the effects of FAAH inhibition upon diclofenac-induced ulceration were not seen in mice lacking cannabinoid-1 receptors^2^. The ability of FAAH inhibition to reduce the ulcerogenic potency of NSAIDs has also been seen with a peripherally-restricted FAAH inhibitor, URB937 (*N*-cyclohexyl-carbamic acid, 3′-(aminocarbonyl)−6-hydroxy[1,1′-biphenyl] − 3-yl ester) and with indomethacin as NSAID^4^. A second endocannabinoid, 2-arachidonoylglycerol (2-AG) is primarily hydrolysed by monoacylglycerol lipase, and inhibition of that enzyme also reduces the ulcerogenic potency of diclofenac^5^^,^^6^.

Taken together, the studies above suggest that a compound with dual-action effects towards both cyclooxygenase (COX, the primary target of NSAIDs) and FAAH (or monoacylglycerol lipase) may be a potentially useful anti-inflammatory agent lacking the problematic gastrointestinal unwanted effects associated with NSAIDs. In 2015, the Piomelli group reported the synthesis and pharmacological properties of ARN2508 ((±)−2-[3-fluoro-4-[3-(hexylcarbamoyloxy)phenyl]phenyl]propanoic acid), a compound combining the structural elements of URB597 and the NSAID flurbiprofen^7^^,^^8^. The compound inhibited FAAH, COX-1 and COX-2 with IC_50_ values of 31, 12 and 420 nM, respectively, and produced anti-inflammatory effects *in vivo* without causing gastric irritation^7^. The carbamate group in the molecule was required for (presumably irreversible) FAAH inhibition, but not for COX-inhibition^5^. Similar to the profens^9^, the compound shows substrate-selective inhibition of COX-2, being more potent when 2-AG is used as substrate than when arachidonic acid (AA) is used^10^.

**Figure 1.**
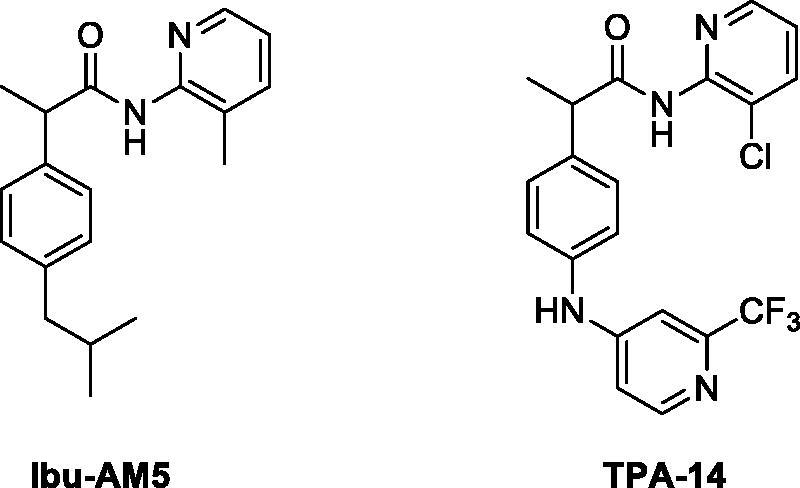
Structure of **Ibu-AM5** and **TPA-14**.

An alternative approach has been to design compounds based on ibuprofen, which has modest FAAH-inhibitory activity^11^, and to optimise the FAAH-inhibitory properties while retaining the COX-inhibitory properties of the parent compound. The first such compound, a heterocyclic amide ibuprofen analoge, **Ibu-AM5** (2–(4-isobutylphenyl)-N-(3-methylpyridin-2-yl)propenamide, Figure 1) had been shown previously by one of us in 2003 to have analgesic activity with respect to acetic acid-induced visceral nociception in the mouse, without appreciable ulcerogenic potency^12^, and successively further described in 2007 for its FAAH inhibitory activity^13^. Further studies by us have shown that the compound inhibits FAAH in a mixed-type manner in sub-micromolar concentrations (i.e. 2-3 orders of magnitude more potent than ibuprofen itself) while retaining the substrate-selective inhibition of COX-2 seen with ibuprofen^14^^,^^15^.

While **Ibu-AM5** is a potentially useful compound, it would be useful to explore its structure to determine whether more potent FAAH/COX dual inhibitors can be identified. SAR studies so far reported by us have^14^^,^^16^^,^^17^, however, been unsuccessful in that the most potent FAAH-inhibitory compound so far described, *N*-(3-chloropyridin-2-yl)−2–(4-((2-(trifluoromethyl)pyridin-4-yl)amino)phenyl)propenamide, **TPA-14** (*N*-(3-chloropyridin-2-yl)−2–(4-((2-(trifluoromethyl)pyridin- 4-yl)amino)phenyl)propenamide, Figure 1), had a similar potency to that of **Ibu-AM5**, but lacked COX-inhibitory potency^16^. In the present study, we report the identification of a novel **Ibu-AM5** analogue that is more potent than **Ibu-AM5** but which retains its substrate-selective inhibition of COX-2.

## Experimental

### Materials

Anandamide [ethanolamine-1-^3^H] ([^3^H]AEA, specific activity 2.22 TBq mmol^−1^) was purchased from American Radiolabeled Chemicals, Inc (St. Louis, MO). Non-radioactive AEA, ovine COX-1 (cat. no. 60100), human recombinant COX-2 (cat. no. 60122), arachidonic acid (AA) and 2-AG were purchased from the Cayman Chemical Co. (Ann Arbour, MI, USA). All commercially available solvents and reagents were used without further purification and were purchased from Sigma-Aldrich (Milan, Italy).

### Chemistry

**Scheme 1.**
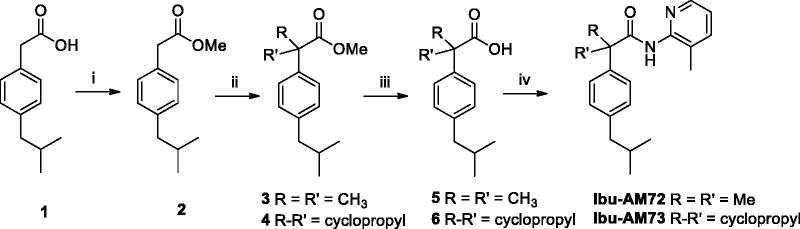
Synthetic pathway for **Ibu-AM72** and **73**. Reagents and conditions: (i) HCl 37%, MeOH, reflux 4 h; (ii) Lithium bis-(trimethylsilyl)amide, THF, −78 °C, 45 min, then MeI or 1,2-dibromoethane, 1 h; (iii) 5 N NaOH, H_2_O, EtOH, r.t., 24 h; (iv) EDC, HOBt, MeCN, r.t. 36 h.

**Scheme 2.**
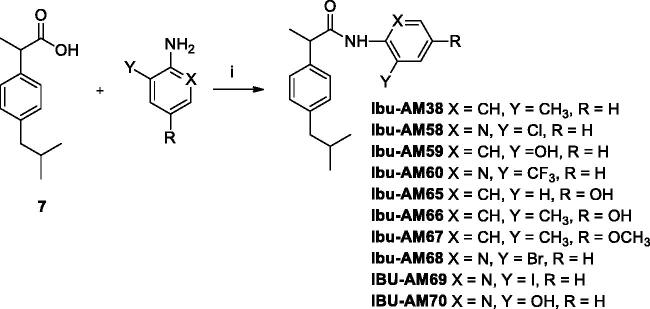
Synthetic pathway for Ibuprofen aryl- and pyridyl-amides. Reagents and conditions i) EDC, HOBt, MeCN, r.t. 36 h.

NMR spectra were recorded on an Inova 500 spectrometer (Varian, Palo Alto, CA). The chemical shifts (δ) are reported in part per million downfield from tetramethylsilane (TMS), which was used as internal standard, and the spectra were recorded in hexadeuteriodimethylsulphoxide (DMSO-d_6_). Infra-red spectra were recorded on a Vector 22 spectrometer (Bruker, Bremen, Germany) in Nujol mulls. The main bands are given in cm^−1^. Positive-ion electrospray ionisation (ESI) mass spectra were recorded on a double-focusing MAT 95 instrument (Finnigan, Waltham, MA) with BE geometry. Melting points (mp) were determined on a SMP1 Melting Point apparatus (Stuart Scientific, Stone, UK) and are uncorrected. All products reported showed ^1^H NMR spectra in agreement with the assigned structures. The purity of the tested compounds was determined by combustion elemental analyses conducted by the Microanalytical Laboratory of the Department of Chemical and Pharmaceutical Sciences of the University of Ferrara with a MT-5 CHN recorder elemental analyser (Yanagimoto, Kyoto, Japan) and the values found were within 0.4% of theoretical values. Ibuprofen amides **Ibu-AM38-73** were synthesised according to Schemes 1 and 2.

### Methyl 2–(4-isobutylphenyl)acetate (2)

A solution of Ibufenac **1** (1.92 g, 10 mmol) in CH_3_OH (10 ml) was added at room temperature (r.t.) with 37% HCl (0.5 ml) and refluxed for 4 h. The solvent was removed under vacuum and the crude methyl ester was used without purification in the further step. Yield 85%. Oil. ^1^H NMR (DMSO-d_6_) δ 0.98 (d, *J* = 7.0 Hz, 6H, CH_3_), 1.93 (*m*, 1H, CH), 2.42 (d, *J* = 7.0 Hz, 2H, CH_2_), 3.65 (*s*, 2H, CH_2_), 3.78 (*s*, 3H, CH_3_), 7.15 (*m*, 2H, Ar), 7.19 (*m*, Hz, 2 H, Ar). Elemental analysis: calculated for C_13_H_18_O_2_ (206.29)% C 75.69; H 8.80; found % C 75.75; H 8.77. Physical and spectral data were in accordance with literature values^18^.

### General procedure for the synthesis of esters 3 and 4

Lithium bis-(trimethylsilyl)amide (4.00 g, 24 mmol) was added to a solution of ester **2** (2.00 g, 9.7 mmol) in dry THF (40 ml) under argon at −78 °C, the mixture was stirred at this temperature for 45 min. Then methyl iodide (3.40 g, 24 mmol) or 1,2-dibromoethane (4.51 g, 12 mmol) was added dropwise to the stirred solution for an additional 1 h. The mixture was poured in water and the desired product was extracted with diethyl ether (2 × 30 ml). The solvent was dried over Na_2_SO_4,_ then it was evaporated under reduced pressure. The crude residue was purified via silica gel (200-400 mesh silica gel Merk KGaA) chromatography using petroleum ether 40–60 °C and AcOEt 20:1.

### Methyl 2–(4-isobutylphenyl)-2-methylpropanoate (3)

Yield 80%. Oil. ^1^H NMR (DMSO-d_6_) δ 0.95 (d, *J* = 7.0 Hz, 6H, CH_3_), 1.63 (*s*, 6H, CH_3_), 1.82 (*m*, 1H, CH), 2.42 (d, *J* = 7.0 Hz, 2H, CH_2_), 3.66 (*s*, 3H, CH_3_), 7.07 (d, *J* = 7.5 Hz, 2H, Ar), 7.25 (d, *J* = 7.5 Hz, 2 H, Ar). Elemental analysis: calculated for C_15_H_22_O_2_ (234.16)% C 76.88; H 9.46; found % C 76.92; H 9.44. Physical and spectral data were in accordance with literature values^19^.

### Methyl 1-(4-isobutylphenyl)cyclopropane-1-carboxylate (4)

Yield 60%. Oil. ^1^H NMR (DMSO-d_6_) δ 0.91 (d, *J* = 7.0 Hz, 6H, CH_3_), 1.25 (*m*, 2H, CH_2_), 1.58 (*m*, 2H, CH_2_), 1.84 (*m*, 1H, CH), 2.43 (d, *J* = 7.0 Hz, 2H, CH_2_), 3.69 (*s*, 3H, CH_3_), 7.15 (d, *J* = 7.5 Hz, 2 H, Ar), 7.23 (d, *J* = 7.5 Hz, 2 H, Ar). Elemental analysis: calculated for C_15_H_20_O_2_ (232.32)% C 77.55; H 8.68; found % C 77.62; H 8.72. Physical and spectral data were in accordance with literature values^19^.

### General procedure for the synthesis of acids 5 and 6

To a solution of ester **3** or **4** (1 mmol) in EtOH (10 ml) 5 N solution of NaOH (2 ml) and water (2 ml) were added. The resulting mixture was stirred at r.t. for 24 h. After removing EtOH under vacuum to the resulting solution ice was added and then acidified with aqueous 20% HCl solution until pH 3–4. The formed precipitate was filtrated, washed with water and re-crystallized from *n*-hexane.

### 2–(4-Isobutylphenyl)-2-methylpropanoic acid (5)

Obtained following the general procedure starting by ester **4**. Yield 90%. m.p. 70-72 °C. ^1^H NMR (DMSO-d_6_) δ 0.90 (d, *J* = 7.0 Hz, 6H, CH_3_), 1.64 (*s*, 6H, CH_3_), 1.90 (*m*, 1H, CH), 2.55 (d, *J* = 7.0 Hz, 2H, CH_2_), 7.09 (d, *J* = 7.5 Hz, 2H, Ar), 7.33 (d, *J* = 7.5 Hz, 2 H, Ar). Elemental analysis: calculated for C_14_H_20_O (220.15)% C 76.33; H 9.15; found % C 76.33; H 9.03. Physical and spectral data were in accordance with literature values^19^.

### 1–(4-Isobutylphenyl)cyclopropane-1-carboxylic acid (6)

Obtained following the general procedure starting by ester **5**. Yield 90%. 74-76 °C. ^1^H NMR (DMSO-d_6_) δ 0.87 (d, J = 7.0 Hz, 6H, CH_3_), 1.08 (m, 2H, CH_2_), 1.41 (m, 2H, CH_2_), 1.80 (*m*, 1H, CH), 2.42 (d, *J* = 7.0 Hz, 2H, CH_2_), 7.06 (d, *J* = 7.5 Hz, 2 H, Ar), 7.21 (d, *J* = 7.5 Hz, 2 H, Ar). Elemental analysis: calculated for C_14_H_18_O_2_ (218.30)% C 77.03; H 8.31; found % C 77.10; H 8.27. Physical and spectral data were in accordance with literature values^19^.

### General procedure for the synthesis of amides Ibu-AM38-73

The solution of the appropriate acid **2**, **5** or **6** (1 mmol), 1-(3-dimethylaminopropyl)−3-ethylcarbodiimide hydrochloride (EDC) (0.19 g, 1.1 mmol) and 1-hydroxybenzotriazole (HOBt) (0.13 g, 1 mmol) in anhydrous MeCN (10 ml) was stirred at r.t., after 30 min the opportune amine (1 mmol) was added and the mixture was stirred at r.t. for 36 h. After the solvent was removed under vacuum. The residue was dissolved in AcOEt (20 ml) and washed sequentially with brine (2 × 5 ml), 10% citric acid (2 × 5 ml), saturated NaHCO_3_ aqueous solution (2 × 5 ml) and water (2 × 5 ml). The organic layer was dried over anhydrous Na_2_SO_4_ and evaporated under vacuum to give the title amides.

### 2–(4-Isobutylphenyl)-N-(o-tolyl)propanamide (Ibu-AM38)

Obtained following the general procedure by the condensation between ibuprofen and 2-methylaniline. Yield 95%. Oil. ^1^H NMR (DMSO-d6) δ 0.85 (d, *J* = 7.0 Hz, 6H, CH_3_), 1.41 (d, *J* = 7.0 Hz, 3H, CH_3_), 1.83 (q, *J* = 7.0 Hz 1H, CH), 2.04 (*s*, 3H, CH_3_), 2.42 (*q*, *J* = 7.0 Hz, 2H, CH_2_), 3.88 (*q*, *J* = 7.0 Hz, 1H, CH), 7.04–7.10 (*m*, 6H, Ar), 7.31 (*m*, 2H, Ar), 9.30 (*s*, 1H, NH). IR (Film) 3265, 2955, 2929, 1659, 1528 cm^−1^. Elemental analysis: calculated for C_20_H_25_NO (295.43)% C 81.31; H 8.53; N 4.74; found % C 81.39; H 8.56; N 4.72.

### N-(3-Chloropyridin-2-yl)-2-(4-isobutylphenyl)propanamide (Ibu-AM58)

Obtained following the general procedure by the condensation between ibuprofen and 2-amino-3-chloropyridine. Yield 39%. Oil. Yield 39%. Oil. ^1^H NMR (DMSO-d_6_) δ 0.85 (d, *J* = 6.5 Hz, 6H, CH_3_), 1.34 (d, *J* = 7.0 Hz, 3H, CH_3_), 1.41 (d, *J* = 7.0 Hz, 2H, CH_2_), 1.82 (*m*, *J* = 7.0 Hz, 1H, CH), 3.88 (*m*, 1H, CH), 7.09–8.37 (*m*, 7H, Ar), 10.25 (*s*, 1H, NH). 13 C NMR (DMSO-d_6_) δ 21.6, 25.4 (2 C), 32.7, 47.3, 116.9, 120.2, 130.1 (2 C), 132.0 (2 C), 142. 6, 143.2, 147.8, 150.1, 160.2, 178.7.IR (Film) 1660, 1512 cm^−1^. Elemental analysis: calculated for C_18_H_21_ClN_2_O (316.83)% C 68.24; H 6.68; N 8.84; found % C 68.30; H 6.65; N 8.81.

### N-(2-Hydroxyphenyl)-2-(4-isobutylphenyl)propanamide (Ibu-AM59)

Obtained following the general procedure by the condensation between ibuprofen and 2-hydroxyaniline. Yield 52%. m.p. 123 − 125 °C. ^1^H NMR (DMSO-d_6_) δ 0.85 (d, *J* = 7.0 Hz, 6H, CH_3_), 1.40 (d, *J* = 6.5 Hz, 3H, CH_3_), 1.82 (hept, *J* = 6.5-7.0 Hz 1H, CH), 2.40 (*q*, *J* = 6.5 Hz, 2H, CH_2_), 4.00 (*q*, *J* = 6.5 Hz, 1H, CH), 6.72 (*m*, 1H, Ar), 6.82 (*m*, 1H, Ar), 6.91 (*m*, 1H, Ar), 7.10 (*m*, 2H, Ar), 7.30 (*m*, 2H, Ar), 7.79 (*m*, 1H, Ar), 9.12 (*s*, 1H, OH), 9.73 (*s*, 1H, NH). IR (Nujol) 3359, 3091, 2733, 1654, 1592 cm^−1^. Elemental analysis: calculated for C_19_H_23_NO_2_ (297.17)% C 76.74; H 7.80; N 4.71; found % C 76.68; H 7.83; N 4.73.

### 2-(4-Isobutylphenyl)-N-(3-(trifluoromethyl)pyridin-2-yl)propanamide (Ibu-AM60)

Obtained following the general procedure by the condensation between ibuprofen and 2-amino-3-(trifluoromethyl)pyridine. Yield 59%. m.p. 112–114 °C. ^1^H NMR (DMSO-d_6_) δ 0.85 (d, *J* = 6.0 Hz, 6H, CH_3_), 1.39 (d, *J* = 7.0 Hz, 3H, CH_3_), 1.81 (q, *J* = 6.5 Hz, 1H, CH), 2.42 (d, *J* = 7.0 Hz, 2H, CH_2_), 3.87 (*q*, *J* = 7.0 Hz, 1H, CH), 7.10–8.71 (*m*, 7H, Ar), 10.25 (*s*, 1H, NH). IR (Nujol) 3253, 1670, 1583 cm^−1^. Elemental analysis: calculated for C_19_H_21_F_3_N_2_O (350.16)% C 65.13; H 6.04; N 8.00; found % C 65.08; H 6.06; N 7.96.

### N-(4-Hydroxyphenyl)-2-(4-isobutylphenyl)propanamide (Ibu-AM65)

Obtained following the general procedure by the condensation between ibuprofen and 4-hydroxyaniline. Yield 47%. m.p. 113–115 °C. ^1^H NMR (DMSO-d_6_) δ 0.85 (d, *J* = 7.0 Hz, 6H, CH_3_), 1.37 (d, *J* = 7.0 Hz, 3H, CH_3_), 1.80 (*q*, *J* = 7.0 Hz, 1H, CH), 2.40 (d, *J* = 7.0 Hz, 2H, CH_2_), 3.72 (*q*, *J* = 7.0 Hz, 1H, CH), 6.66 (d, *J* = 8.5 Hz, 2H Ar), 7.01 (d, *J* = 8.0 Hz, 2H, Ar), 7.28 (d, *J* = 8.0 Hz, 2H, Ar), 7.35 (d, *J* = 8.5 Hz, 2H, Ar), 9.14 (*s*, 1H, OH), 9.77 (*s*, 1H, NH). IR (Nujol) 3299, 1653, 1609, 1538 cm^−1^. Elemental analysis: calculated for C_19_H_23_NO_2_ (297.17)% C 76.74; H 7.80; N 4.71; found % C 76.80; H 7.77; N 4.73.

### N-(4-Hydroxy-2-methylphenyl)-2-(4-isobutylphenyl)propanamide (Ibu-AM66)

Obtained following the general procedure by the condensation between ibuprofen and 4-hydroxy-2-methylaniline. Yield 63%. m.p. 133–135 °C. ^1^H NMR (DMSO-d_6_) δ 0.83 (d, *J* = 7.0 Hz, 6H, CH_3_), 1.35 (d, *J* = 7.0 Hz, 3H, CH_3_), 1.77 (hept, *J* = 7.0 Hz, 1H, CH), 1.90 (*s*, 3H, CH_3_), 2.40 (d, *J* = 7.0 Hz, 2H, CH_2_), 3.75 (*q*, *J* = 7.0 Hz, 1H, CH), 6.48–7.28 (*m*, 7H, Ar), 9.09 (*s*, 1H, OH). IR (Nujol) 3398, 3292, 1656, 1610 cm^−1^. Elemental analysis: calculated for C_20_H_25_NO_2_ (311.43)% C 77.14; H 8.09; N 4.50; found % C 77.06; H 8.11; N 4.52.

### 2-(4-Isobutylphenyl)-N-(4-methoxy-2-methylphenyl)propanamide (Ibu-AM67)

Obtained following the general procedure by the condensation between ibuprofen and 4-methoxy-2-methylaniline. Yield 49%. m.p. 100–102 °C. ^1^H NMR (DMSO-d_6_) δ 0.85 (d, *J* = 7.0 Hz, 6H, CH_3_), 1.40 (d, *J* = 7.0 Hz, 3H, CH_3_), 1.81 (hept, *J* = 7.0 Hz, 1H, CH), 1.99 (*s*, 3H, CH_3_), 2.42 (d, *J* = 7.0 Hz, 2H, CH_2_), 3.70 (*s*, 3H, CH_3_), 3.81 (d, *J* = 7.0 Hz, 1H, CH), 6.69-7.31 (*m*, 7H, Ar), 9.21 (s, 1H, NH). IR (Nujol) 3298, 1655, 1613 cm^−1^. Elemental analysis: calculated for C_21_H_27_NO_2_ (325.45)% C 77.50; H 8.36; N 4.30; found % C 77.56; H 8.39; N 4.28.

### N-(3-Bromopyridin-2-yl)-2-(4-isobutylphenyl)propenamide (Ibu-AM68)

Obtained following the general procedure by the condensation between ibuprofen and 2-amino-3-bromopyridine. Yield 67%. Oil. ^1^H NMR (DMSO-d_6_) δ 0.85 (d, *J* = 6.0 Hz, 6H, CH_3_), 1.34 (d, *J* = 7.5 Hz, 3H, CH_3_), 1.42 (d, *J* = 7.0 Hz, 2H, CH_2_), 1.81 (*m*, 1H, CH), 3.86 (*q*, *J* = 7.0 Hz, 1H, CH), 7.1–8.41 (*m*, 7H, Ar), 10.22 (*s*, 1H, NH). 13 C NMR (DMSO-d_6_) δ 21.7, 25.3 (2 C), 32.7, 47.4, 106.3, 116.7, 130.2 (2 C), 132.1 (2 C), 141.7, 142.6, 143.2, 150.1, 159.4, 178.7. IR (Film) 3240, 1703, 1580 cm^−1^. Elemental analysis: calculated for C_18_H_21_BrN_2_O (360.08)% C 59.84; H 5.86; N 7.75; found % C 59.90; H 5.84; N 7.79.

### N-(3-Iodopyridin-2-yl)-2-(4-isobutylphenyl)propanamide (Ibu-AM69)

Obtained following the general procedure by the condensation between ibuprofen and 2-amino-3-iodopyridine. Yield 53%. Oil. ^1^H NMR (DMSO-d_6_) δ 0.85 (d, *J* = 7.0 Hz, 6H, CH_3_), 1.42 (d, *J* = 7.0 Hz, 3H, CH_3_), 1.80 (hept, *J* = 7.0 Hz, 1H, CH), 2.41 (*m*, 2H, CH_2_), 3.84 (*q*, *J* = 7.0 Hz, 1H, CH), 7.02–8.40 (*m*, 7H Ar), 10.18 (*s*, 1H, NH). IR (Film) 3233, 1675, 1574 cm^−1^. Elemental analysis: calculated for C_18_H_21_IN_2_O (408.28)% C 52.95; H 5.18; N 6.86; found % C 53.01; H 5.16; N 6.90.

### N-(3-Hydroxypyridin-2-yl)-2-(4-isobutylphenyl)propenamide (Ibu-AM70)

Obtained following the general procedure by the condensation between ibuprofen and 2-amino-3-hydroxypyridine. Yield 38%. Oil. ^1^H NMR (DMSO-d_6_) δ 0.85 (d, *J* = 7.0 Hz, 6H, CH_3_), 1.42 (d, *J* = 7.5 Hz, 3H, CH_3_), 1.79 (hept, *J* = 7.0 Hz, 1H, CH), 2.40 (d, *J* = 7.0 Hz, 2H, CH_2_), 3.62 (*q*, *J* = 7.0 Hz, 1H, CH), 7.09–7.88 (*m*, 7H, Ar), 10.24 (*s*, 1H, NH), 10.75 (*s*, 1H, OH). IR (Film) 1662, 1512 cm^−1^. Elemental analysis: calculated for C_18_H_22_N_2_O_2_ (298.39)% C 72.46; H 7.43; N 9.39; found % C 72.54; H 7.45; N 9.42.

### 2-(4-Isobutylphenyl)-2-methyl-N-(3-methylpyridin-2-yl)propanamide (Ibu-AM72)

Obtained following the general procedure by the condensation between 2-(4-isobutylphenyl)−2-methylpropanoic acid (5) and 2-amino-3-methylpyridine. Yield 70%. Oil. ^1^H NMR (DMSO-d_6_) δ 0.86 (d, *J* = 7.0 Hz, 6H, CH_3_), 1.57 (*s*, 6H, CH_3_), 2.02 (*m*, 1H, CH), 2.03 (*s*, 3H, CH_3_), 2.44 (d, *J* = 7.0 Hz, 2H, CH_2_), 7.12–7.20 (*m*, 3H, Ar), 7.30–7.35 (*m*, 2H, Ar), 7.62 (*m*, 1H, Ar), 8.22 (*m*, 1H, Ar), 9.27 (*s*, 1H, NH). IR (Film) 3167, 2956, 1686, 1583 cm^−1^. Elemental analysis: calculated for C_20_H_26_N_2_O (310.44)% C 77.38; H 8.44; N 9.02; found % C 77.31; H 8.47; N 9.06.

### 3-Methylpyridin-2-yl 1–(4-isobutylphenyl)cyclopropane-1-carboxylate (Ibu-AM73)

Obtained following the general procedure by the condensation between 1–(4-isobutylphenyl)cyclopropane-1-carboxylic acid (6) and 2-amino-3-methylpyridine.Yield 73%. Oil.1H NMR (DMSO-d_6_) δ 0.87 (d, *J* = 7.0 Hz, 6H, CH_3_), 1.10 (*m*, 2H, CH_2_), 1.44 (*m*, 2H, CH_2_), 1.82 (*m*, 1H, CH), 2.03 (*s*, 3H, CH_3_), 2.42 (d, *J* = 7.0 Hz, 2H, CH_2_), 7.07–7.25 (*m*, 4H, Ar), 7.37 (*m*, 1H, Ar), 7.62 (*m*, 1H, Ar), 8.19 (*m*, 1H, Ar), 8.59 (*s*, 1H, NH). IR (Film) 3397, 1687, 1582 cm^−1^. Elemental analysis: calculated for C_20_H_24_N_2_O (308.43)% C 77.89; H 7.84; N 9.08; found % C 77.82; H 7.87; N 9.04.

### Pharmacology

#### FAAH assay

Frozen (−80 °C) brains (minus cerebella) from adult Wistar or Sprague-Dawley rats were thawed and homogenised in 20 mM HEPES, 1 mM MgCl_2_, pH 7.0. The homogenates were then centrifuged at ∼35000 x g for 20 min at 4 °C followed by washing (by recentrifugation and by resuspension in the buffer) twice before incubation at 37 °C for 15 min in order to hydrolyse all endogenous FAAH substrates. After a further centrifugation, pellets were resuspended in 50 mM Tris-HCl buffer, pH 7.4, containing 1 mM EDTA and 3 mM MgCl_2_, and frozen at −80 °C in aliquots until used for the assay. For the FAAH assay^20^, test compounds, homogenates and [^3^H]AEA (diluted with non-radioactive AEA to give a substrate concentration of 0.5 μM) in 10 mM Tris- HCl, 1 mM EDTA, pH 7.4, containing 1% w/v fatty acid-free bovine serum albumin were incubated for 10 min at 37 °C. Activated charcoal in 0.5 M HCl was added to adsorb the unmetabolized [^3^H]AEA and the samples were mixed and left at r.t. for ∼30 min. Following centrifugation at 2000 *g* for 10 min, aliquots of the supernatants, containing the [^3^H]ethanolamine produced by hydrolysis of [^3^H]AEA, were analysed for tritium content by liquid scintillation spectroscopy with quench correction. Blank values were obtained by the use of buffer rather than homogenate.

Data were expressed as % of vehicle (ethanol) control and analysed using the algorithm log(inhibitor) vs. response – variable slope (four parameters) built into the GraphPad Prism computer programme v8.3 for the Macintosh (GraphPad Software Inc., San Diego, CA). The programme reports 95% confidence limits (profile likelihood) for the IC_50_ values and these presented in the results.

#### COX-1 and 2 assay

The assay was performed essentially according to the method of Meade et al^21^. An oxygen electrode chamber with integral stirring (Oxygraph System, Hansatech Instruments, King ´s Lynn, U.K.) was calibrated daily to ambient temperature and air pressure. The assay buffer contained 0.1 M Tris-HCl buffer pH 7.4, 1 μM haematin, 2 mM phenol, 5 mM EDTA, 10 μM substrate (AA or 2-AG) in a final assay volume was 2 ml. After addition of test compound, a baseline was established for 5 min before initiation of reaction by addition of 200 units ovine COX-1 or human recombinant COX-2. The change in oxygen consumption as a measurement of enzyme activity was monitored for approximately 5 min.

### Computational studies

#### FAAH receptor and ligand preparation

The crystal structure of the rat fatty acid amide hydrolase (rFAAH) (PDB ID: 3QK5) was downloaded from the Protein Data Bank website. Both monomers A and B were treated with the Protein Preparation Wizard^22^ tool implemented in Maestro ver. 11.12^23^, in order to add all the hydrogen atoms and assign the correct bond orders. Subsequently, both the co-crystallized ligands and water molecules were removed. Residue Lys142 was considered in its deprotonated form, according to the proposed catalytic mechanism of FAAH^24–26^. The 3 D structure of **Ibu-AM68** was built using the Graphical User Graphical User Interface (GUI) of Maestro ver. 11.12^23^. The protonation state of **Ibu-AM68** at pH 7.4 in water has been calculated using the Epik module^27^. Finally, **Ibu-AM68** was then minimised using a protocol already adopted for **Ibu-AM5**:^17^ OPLS 2005 force field using the Polak-Ribiere Conjugate Gradient (PRCG)^28^ algorithm and 2500 iteration steps.

#### Docking of Ibu-AM68 in FAAH

The molecular docking of **Ibu-AM68** was performed only on the monomer A of the rat FAAH (rFAAH) receptor. Docking procedure was carried out with the Glide software package^29^, using the Standard Precision (SP) algorithm of the GlideScore function^30^^,^^31^ and the OPLS 2005 force field^32^. A grid box of 29 × 29 × 29 Å centred on the ligand binding cavity was created. A total amount of 200 poses was generated and the conformational sampling of the ligand was enhanced by two times, as reported by the default setting of Glide. Docking conformations of **Ibu-AM68** were then clusterised based on their RMSD cut-off of 2 Å. Globally, ten clusters were obtained and, among them, only the conformation included in the most populated cluster owing both the Glide Emodel and GlideScore lowest-energy value was considered (Figure 4). Such conformation was, finally, submitted to a further minimisation protocol using the OPLS 2005 force field^32^, 20,000 minimisation steps and the Polak-Ribiere Conjugate Gradient (PRCG) algorithm^28^.

## Results and discussion

The potency of **Ibu-AM5** towards AEA hydrolysis has been measured by different groups with different assay methodologies, FAAH preparations (rat brain, mouse brain, recombinant human FAAH) and substrate concentrations (0.5–2 µM)^13–15^^,^^33^^,^^34^. The IC_50_ value for **Ibu-AM5** from different studies in our laboratory using the same assay as here (of importance given the mixed-type nature of its inhibition of FAAH) ranges from 0.52 − 1.2 µM^14^^,^^15^ and we therefore have used the most potent value for comparative purposes, since the aim is to identify more potent compounds.

### FAAH inhibition

**Figure 2.**
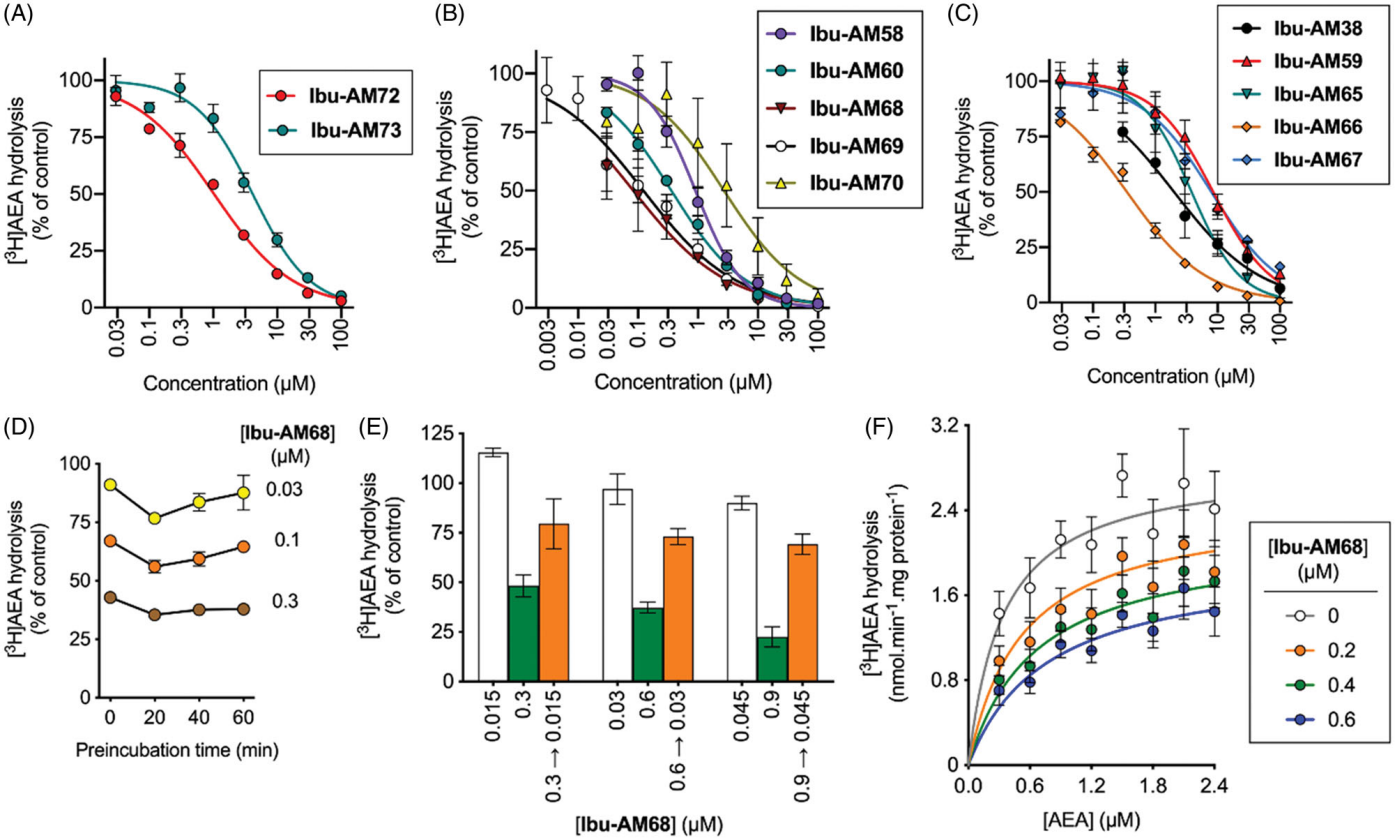
Inhibition of [^3^H]AEA hydrolysis by analogues of **Ibu-AM5**. (A–C) show concentration-response curves for the inhibition by the compounds shown of the hydrolysis of 0.5 µM [^3^H]AEA by rat brain homogenates. In (D), the homogenates were preincubated with **Ibu-AM68** for the times shown prior to addition of 0.5 µM [^3^H]AEA. In €, rat homogenates (at 20-fold normal strength) were preincubated with either vehicle, 0.3, 0.6 or 0.9 µM **Ibu-AM68** for 60 min. Aliquots were then diluted 20-fold and assayed for FAAH activity with 0.5 µM [^3^H]AEA. These are shown as 0.3 → 0.015, 0.6 → 0.03 and 0.9 → 0.045 in the figure. Concomitantly, **Ibu-AM68** was added to vehicle-preincubated aliquots to give concentrations of 0.015, 0.03 and 0.045 µM (representing free concentrations after a 20-fold dilution), 0.3, 0.6 and 0.9 µM final concentrations. (F) shows the kinetics of the inhibition of rat FAAH by **Ibu-AM68**. The data was better fitted by a mixed-type inhibition mode of inhibition (K_i_ value of 0.26 µM and an α value of 4.9) than by a competitive mode of inhibition. In (A–D), data are means ± SEM (when not enclosed by the symbols), N = 3, except for the data for **Ibu-AM69** in Figure 1(B) where N = 3-7. In (D), data are means ± SEM, N = 4.

Three series of **Ibu-AM5** analogues were synthesised according to Schemes 1 and 2 and tested towards rat brain FAAH-catalysed hydrolysis of AEA. The first series of two compounds was motivated by the finding that for **Ibu-AM5** removal of the methyl group at the C-2 carbon atom (“Ibufenac-AM1”) reduced the potency roughly 60-fold^15^. In order to explore whether or not the methyl group at that position was optimal, two compounds were synthesised, with a dimethyl (**Ibu-AM72**) and cyclopropyl (**Ibu-AM73**) groups instead of the methyl group at the C-2 carbon atom. The amides were obtained starting from Ibufenac (**1**) that was converted into its methyl ester **2** and then alkylated at C-2 position to give intermediates **3** and **4** that were hydrolysed to the corresponding acid **5** and **6**. These last were coupled with 2-amino-3-methylpyridine by EDC method to afford the target amides **Ibu-AM72** and **Ibu-AM73**. The compounds inhibited FAAH with IC_50_ values of 1.0 and 4.1 µM for **Ibu-AM72** and **Ibu-AM73**, respectively (Figure 2A, Table 1). Although this is a very limited series, it would suggest that there is little to be gained by adjusting the methyl group at the C-2 carbon atom, and so we moved on to the amido moiety of **Ibu-AM5** 3-substituent on the pyridine ring of **Ibu-AM5**.

**Table 1.** IC_50_ values for the inhibition by novel Ibu-AM compounds of the hydrolysis of 0.5 µM [^3^H]AEA by rat brain homogenates.

Compound	IC_50_ (µM)	95% confidence limits of the IC_50_
Ibu-AM72	1.0	0.86–1.2
Ibu-AM73	4.1	3.2–5.3
Ibu-AM38	2.0	1.3–3.0
Ibu-AM59	8.5	6.2–12
Ibu-AM65	3.8	2.3–6.5
Ibu-AM66	0.35	0.29–0.42
Ibu-AM67	7.9	5.9–11
Ibu-AM58	0.91	0.72–1.1
Ibu-AM68	0.083	0.038–0.15
Ibu-AM69	0.12	0.078–0.19
Ibu-AM60	0.36	0.32–0.41
Ibu-AM70	2.7	1.1–6.4

In our initial study^12^, we reported that the potency of the pyridinamides of ibuprofen towards FAAH is related to the presence of both methyl and pyridine nitrogen in ortho positions to the amide nitrogen as in **Ibu-AM5**, the methyl absence or its moving in a position different from ortho to the amide nitrogen results in activity decrease. On this basis to evaluate the influence on the activity of the pyridine nitrogen, we changed pyridine ring with a phenyl. With this purpose, we prepared the 2-methylphenyl analogue of **Ibu-AM5**. The replacement of pyridine ring with a phenyl as in **Ibu-AM38** produced activity decrease (IC_50_ 2.0 μM). After further evaluation a group able to establish hydrogen bond with the enzyme was inserted on the amide phenyl ring. With this purpose, the 2-hydroxy (**Ibu-AM59**) and the 4-hydroxy (**Ibu-AM65**) derivatives were designed. As indicated by their IC_50_ values, the presence of the hydroxy group in 4-position caused an increase in activity (3.8 µM for **Ibu-AM65** vs 8.5 µM for **Ibu-AM59**), although these compounds were among the least potent in the series. Next step was the integration of this activity enhancement with the presence of a 2-methyl group, with this purpose the compound **Ibu-AM66** was prepared by condensation of ibuprofen with 2-methyl-4-hydroxyaniline. **Ibu-AM66** showed very good activity with an IC_50_ value of 0.35 μM. With the aim to study, the influence on the inhibitory activity of hydrogen bond donor or acceptor group **Ibu-AM66** was modified by replacement of the 4-hydroxy by a methoxy group to afford **Ibu-AM67** (IC_50_ 4.6 μM). The result was decrease in activity, indicating that a hydrogen bond donor is better than an acceptor. Thereafter, we focussed on alternative substituents to the 3-methyl group such us halogens, trifluoromethyl and hydroxy groups. The data for these amides are shown in Figure 2(B) and summarised in Table 1. The observed potencies of the substituents were -I (**Ibu-AM69**) and -Br (**Ibu-AM68**) > -CF_3_ (**Ibu-AM60**) > -Cl (**Ibu-AM58**) > -OH (**Ibu-AM70**). The 95% confidence intervals for the mean IC_50_ values for **Ibu-AM69** (0.078–0.19 µM) and **Ibu-AM68** (0.038–0.15) overlap, so we regard the two compounds as equipotent but more potent than **Ibu-AM5**.

The inhibition of FAAH by **Ibu-AM68** was investigated in more detail. Preincubation of **Ibu-AM68** with the homogenates for up to 60 min prior to addition of substrate did not increase the observed inhibition, indicating that there is no time-dependence of the inhibition (Figure 2(D)). For a fully reversible inhibitor, preincubation for 60 min with a concentration “x” of compound followed by a 20-fold dilution prior to addition of substrate should produce the same observed inhibition as seen with a concentration of “x/20” of the compound added together with the substrate, and this was found to be the case for **Ibu-AM68** (Figure 2(E)). Finally, kinetic experiments indicated a simple mixed-model inhibition of FAAH with a K_i_ value of 0.26 µM and an α value (the ratio of the K_i_^intercept^: K_i_^slope^ values; for pure competitive inhibition, α→ ∞) of 4.9 (Figure 2(F)). Thus, **Ibu-AM68** is a reversible mixed inhibitor of rat brain FAAH with a greater potency than **Ibu-AM5**.

### Inhibition of COX isoenzymes by Ibu-AM68

**Figure 3.**
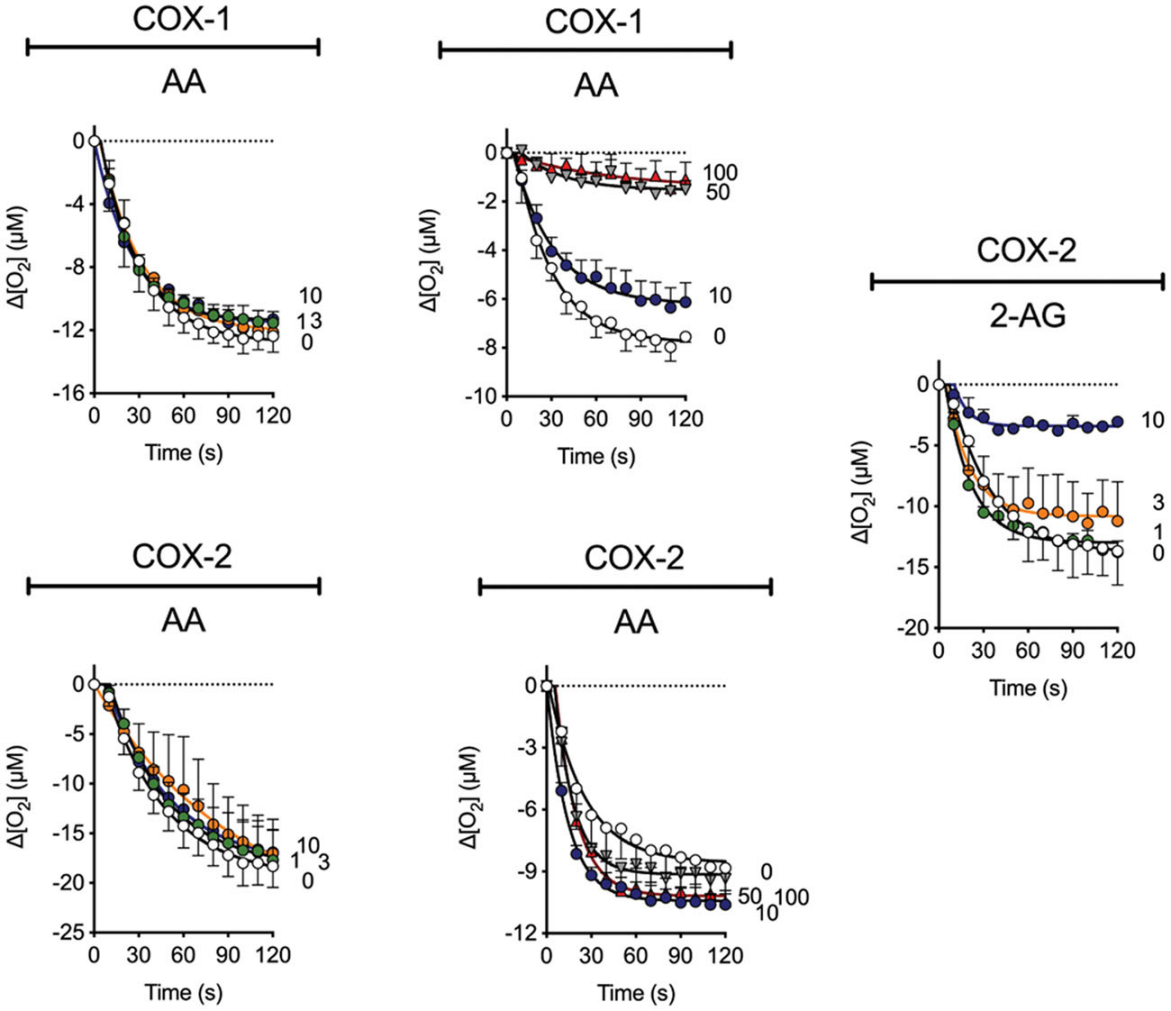
The influence of **Ibu-AM68** upon the cyclooxygenation of 10 µM arachidonic acid (AA) and 2-arachidonoylglycerol (2-AG) by COX-1 and COX-2. Shown are means ± SEM, N = 3 in each graph for the changes in oxygen tension following addition of enzyme in the presence of **Ibu-AM68**. The concentrations of **Ibu-AM68**, in µM, are shown on the right of each panel. The enzyme isoform and substrate used is given above each panel. Note that there are two different panels for COX-1 and AA and for COX-2 and AA. This was because the experiments were performed on different occasions with different batches of the enzyme. COX-1 does not metabolise 2-AG.

The ability of **Ibu-AM68** to inhibit the cyclooxygenation of AA and 2-AG by COX-1 and COX-2 was investigated (Figure 3). In our hands under the assay conditions used, 30 µM ibuprofen itself produces approximately 50% inhibition of the cyclooxygenation of AA by COX-1 with at best minor inhibition of COX-2 at this concentration. However, ≥10 and 30 µM ibuprofen produce a marked inhibition of 2-AG and AEA cyclooxygenation by COX-2 (neither endocannabinoid is a substrate for COX-1)^14^^,^^35^. **Ibu-AM5** also shows substrate selective inhibition, reducing the rate of cyclooxygenation to about half at concentrations of 50 µM (COX-1, AA as substrate) and 3 µM (COX-2, AEA as substrate) whilst 100 µM **Ibu-AM5** is without effect upon COX-2 with AA as substrate.^14^ At a concentration of 10 µM, a modest inhibition of AA cyclooxygenation by **Ibu-AM68** was seen with COX-1 whereas the cyclooxygenation of 2-AG by COX-2 was almost completely inhibited. Higher concentrations of **Ibu-AM68** (50 and 100 µM) produced a complete inhibition of COX-1 but did not inhibit AA cyclooxygenation by COX-2. This suggests that the substrate-selective inhibition of COX-2 reported first for the *R*-profens by Marnett and colleagues^35^ is also seen with **Ibu-AM68**. The mechanism of this inhibition has not been investigated, but Marnett et al.^9^^,^^36^ have suggested that it may be related to COX-2 (which has a homodimeric structure) acting as functional heterodimers, whereby the binding of the *R*-profen to one site acts allosterically to block 2-AG but not AA cyclooxygenation. It is possible that such a mechanism can explain the actions of **Ibu-AM5** and **Ibu-AM68**.

### FAAH docking on Ibu-AM68

**Figure 4.**
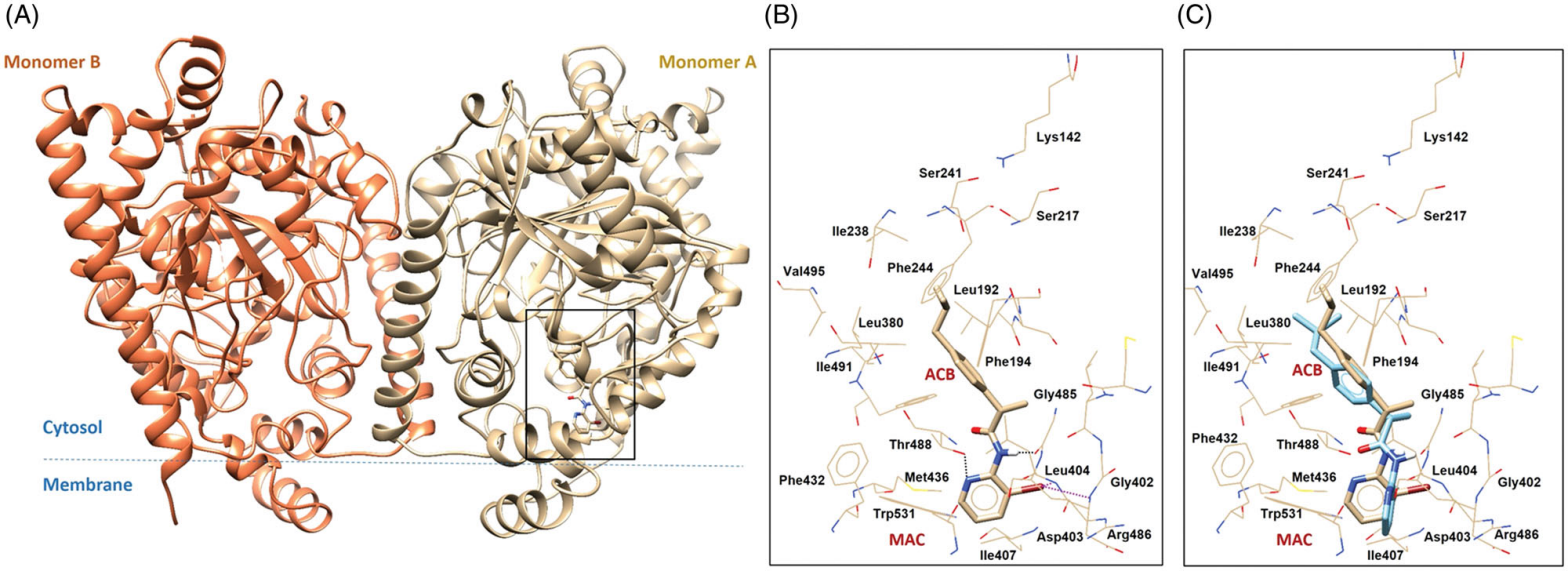
(A) 3 D structure of monomers A and B of rFAAH. The rectangular box indicates the **Ibu-AM68** ligand binding cavity. (B) Focus on the binding mode of **Ibu-AM68** within the ACB channel. Polar contacts engaged by bromine atom with Leu404 and Asp403 are depicted as magenta dashed lines, while hydrogen bond interactions with Gly485 and Thr488 are shown as dashed black lines. (C) Overlap between the binding mode of **Ibu-68AM** (tan stick) and **Ibu-AM5** (light cyan stick), highlighting the different orientation of the substituted pyridine ring.

Molecular docking calculations on (*S*)-**Ibu-AM68** were performed with the Glide software^29–31^ in the crystal structure of rat FAAH (PDB ID: 3QK5)^37^. The software Glide was chosen since it showed to be able to well reproduce the binding poses of (*R*)- and (*S*)- **Ibu-AM5** resulting by molecular dynamics and free energy calculations 0.17 The results were clustered and successively ranked according to the Glide Emodel and the Glide Score. The best pose showed the isobutyl moiety pointing to the catalytic triad and the pyridine moiety entering the membrane access channel (MAC) channel (Figure 4). In particular, the substituted pyridine ring established hydrophobic contacts with Leu404, Ile407, a T-shaped π-π interaction with Trp531 and a H-bond interaction with the hydroxyl group of Thr488. Moreover, polar contacts between the bromine atom and the backbone hydrogens of residues Asp403 and Leu404 were observed. An additional H-bond was established between the NH group of the ligand with the carbonyl of the Gly485. The **Ibu-AM68** hydrophobic isobutyl-phenyl moiety resulted embedded in a hydrophobic region in the Acyl Chain Binding channel (ACB), and established hydrophobic contacts with residues Leu192 Phe244, Leu380, Thr488 and Ile491. The comparison with the binding mode of **Ibu-AM5** showed high similarity in the isobutyl-phenyl moiety, but a different conformation of the pyridine ring with respect to the amide moiety. This different conformational behaviour maybe be likely due to different dipole alignment, being the slightly negative bromine atom better aligned with the NH group of the amide bond, while the methyl substituent preferred an orientation orthogonal to the carbonyl group.

In conclusion, the present study has characterised *in vitro* an **Ibu-AM5** analogue that is slightly more potent than **Ibu-AM5** itself as FAAH inhibitor and which retains its COX-2 substrate-selectivity. Further studies are necessary to determine whether this compound behaves like the dual action FAAH-COX inhibitor ARN2508 in producing potentially beneficial effects in models of inflammatory pain without the ulcerogenic effects that are an issue with current NSAIDs.
